# Assessment of Nutritional Trends and Program Implementation Under the Nutrition Improvement Program

**DOI:** 10.3390/nu18132195

**Published:** 2026-07-06

**Authors:** Huihui Huang, Fei Peng, Xuefeng Yang, Maowei Cheng

**Affiliations:** 1 Department of School Health, Hubei Provincial Center for Disease Control and Prevention, Wuhan 430079, China; pf_hbcdc@163.com; 2 Department of Nutrition and Food Hygiene, Hubei Key Laboratory of Food Nutrition and Safety, MOE Key Laboratory of Environment and Health, School of Public Health, Tongji Medical College, Huazhong University of Science and Technology, Wuhan 430030, China; d202482033@hust.edu.cn

**Keywords:** nutritional improvement program, children and adolescents, nutritional evaluation, malnutrition

## Abstract

**Background:** The Nutrition Improvement Program has been implemented in China for over a decade; however, its assessment in Hubei Province has not yet been systematically reported. This study evaluated the implementation outcomes of the Rural Compulsory Education Students’ Nutrition Improvement Program in Hubei Province. **Methods:** A multi-stage cluster sampling design with stratification by school type was used. Standardized questionnaires were administered to schools, districts, and students to assess the food environment, nutrition policies, and dietary behaviors. Anthropometric and biochemical measurements were collected from all participants in 2014 and 2023. **Results:** A total of 24 schools across six counties in Hubei Province were surveyed at two time points, comprising 8619 students (4388 in 2014 and 4231 in 2023). Between 2014 and 2023, the proportion of students in the Nutrition Improvement Program reporting satisfaction with school meals increased significantly, and average nutritional knowledge scores improved. Nevertheless, several deficiencies persisted: menus remained poorly aligned with dietary guidelines, food variety was limited, full-time nutrition or health teachers were scarce, and absolute levels of nutritional knowledge remained low. In 2023, the prevalence of undernutrition, overweight/obesity, and anemia among program participants was 9.5%, 15.9%, and 12.2%, respectively. Compared with girls, boys had higher rates of undernutrition and overweight/obesity and a lower anemia rate; the same pattern was observed in primary school students relative to junior high school students. Compared with 2014, the nutritional profile shifted markedly (*p* < 0.001). Undernutrition and anemia declined by 7.4 and 4.9 percentage points, respectively, whereas overweight/obesity increased by 6.9 percentage points. **Conclusions:** From 2014 to 2023, students in the Nutrition Improvement Program of Hubei Province experienced observable changes in nutritional status: the primary nutritional concern had shifted from undernutrition to overnutrition, and the prevalence of anemia has generally decreased, while it has increased in some areas. However, given the observational nature of the repeated cross-sectional design, causal inference regarding the program’s impact was not supported.

## 1. Introduction

On 25 February 2021, China officially declared a comprehensive victory in the fight against poverty [1]. This milestone marked the historic eradication of absolute poverty in China and achieved the poverty reduction target of the United Nations 2030 Agenda for Sustainable Development a full decade ahead of schedule [2]. Gaps between urban and rural areas in terms of infrastructure, residents’ income, and social identity have significantly narrowed in recent years. Nevertheless, substantial disparities persist, particularly in the quality of public services, the accumulation of family assets, and the less visible but deeply felt entitlements that shape children’s long-term development, most notably in healthcare, nutrition, and human capital investment [3,4].

For a long time, significant nutritional disparities have existed between urban and rural areas in China. Rural children have lagged considerably behind their urban counterparts in nutritional intake. Globally, particularly in many developing countries, child malnutrition remains a serious issue that hinders socioeconomic progress [5,6]. To improve the nutritional status and overall health of rural students while accelerating the development of rural education and promoting educational equity, the General Office of the State Council promulgated the “Nutrition Improvement Program for Rural Compulsory Education Students” (NIP) in 2011, launching a pilot intervention in contiguous areas of extreme rural disadvantage. Central government subsidies were initially set at 3 Chinese Yuan (CNY) per student per day for 200 school days; the rate was increased to 4 CNY in 2014 and to 5 CNY in 2021 [7]. By 2021, the program covered over 40 million students, accounting for 42.4% of all rural students receiving compulsory education, making it the third largest program of its kind in the world. From 2011 to 2021, the central government invested nearly 197 billion yuan (approximately 31.2 billion US dollars), representing the second largest public investment globally. All provinces and municipalities have actively implemented various components of the NIP. Concurrently, improvements in nutritional status and physical fitness among rural students have been documented [8]. However, some regions still face challenges such as lax food safety management, non-standard use and management of funds, and poor quality of meal provision [9].

In addition to China, many other countries have also implemented school nutrition programs. In India, a nationally mandated school meal program has increased children’s daily nutritional intake by 49% to 100% [10]. In the United States, the National School Lunch Program has shown that free and reduced-price lunches can improve children’s health, reducing food insecurity by at least 6%, improving health conditions by 33%, and decreasing obesity rates by 21% [11]. A large-scale school meals program in Ghana improved the height-for-age of school-age children (5 to 15 years old) from poor families [12]. Despite these international successes, the effectiveness of such programs varies substantially across regions, underscoring the need for context-specific evaluations. Within Hubei Province, the NIP covers 26 state-designated surveillance sites, located primarily in the mountainous northeast, northwest, and southwest. The 2021 roster comprised 1658 primary schools and 300 junior secondary schools, serving 360,649 students. Annual central transfers to the province rose from 220 million CNY in 2011 to 370 million CNY in 2021.

Although previous studies have indicated positive impacts resulting from the implementation of nutrition programs in China, the diets of some children are gradually shifting towards high-fat intake, with a decrease in carbohydrate-derived energy and an increase in fat consumption [13]. In recent years, childhood obesity has attracted widespread attention, placing a significant burden on the country’s healthcare system [14,15,16]. Some studies have also indicated significant regional, gender, and age differences in the rate of growth retardation among children and adolescents under the NIP framework [17,18].

Despite more than a decade of implementation, systematic evaluations of the NIP remain limited, and there is no consensus on a standardized assessment approach [6]. Furthermore, most existing studies have focused on national or western regional data, with limited evidence from central Chinese provinces such as Hubei, where geographic and socioeconomic conditions differ markedly from other regions. This study aims to provide a repeated cross-sectional descriptive assessment of nutritional trends among participants in Hubei Province, serving as a basis for future evaluation framework development. By leveraging Hubei Province’s annual program surveillance data, this study undertakes a comprehensive cross-sectional and repeated cross-sectional assessment of implementation outcomes, identifies key constraints and their determinants, assesses the extent to which student growth lags behind reference standards, and delineates a context-specific evaluation methodology and benchmark set. The resulting evidence base will inform performance-based management of the program by relevant government agencies.

## 2. Methods

### 2.1. Study Design and Participants

A multi-stage stratified cluster sampling was employed. Six pilot counties in Hubei Province were selected in 2014 and 2023. These counties were randomly selected from three geographic strata (southwest, northwest, and northeast Hubei) among all officially designated NIP implementation sites. Stratification also considered baseline anemia prevalence rates to ensure representation of counties with varying nutritional risk levels. All selected counties were classified as poverty-stricken at the time of sampling. And the 2 surveys were conducted as two independent cross-sectional investigations, both recruiting students from the same schools and geographic regions. Within each county, four schools (two primary and two junior-secondary) were randomly selected. In every sampled school, one or two intact classes per grade were randomly selected. Each selected class contained no fewer than 40 students, with approximate gender balance. Accordingly, 240 students per primary school and 120 students per junior secondary school were enrolled, resulting in a total of 24 schools across the six counties. In addition, blood samples were collected from students to test the levels of total blood hemoglobin. All investigators received standardized training prior to data collection, and all participants or their guardians provided written informed consent. The study was approved by the Ethics Review Committee of Hubei Provincial Center for Disease Control and Prevention (Ethical approval code: 2023-004-02). The ethics approval date for our study is 31 May 2023.

### 2.2. Field Investigation

A comprehensive field survey was conducted at two levels: county and school. County-level data documented the socioeconomic context and overall implementation progress of the NIP across the six target counties. Using self-developed questionnaire, school-level data were collected from all 24 participating primary and secondary schools, covering the following aspects: program implementation fidelity, canteen infrastructure and staffing, meal provision efficiency (including food variety, procurement sources, and alignment of menus with local students’ nutritional requirements), and delivery of health education curricula.

Within each school, one intact class was randomly selected from Grade 4 and Grade 5 of primary school and from Grade 1 and Grade 2 of junior-secondary school. Students in these classes completed structured questionnaires covering sociodemographic characteristics, validated measures of nutritional knowledge, and satisfaction with school meals provided under the Program. The internal consistency and construct validity of the questionnaire were assessed. The Cronbach’s α coefficient was 0.652, indicating moderate internal consistency. The KMO value was 0.77, and Bartlett’s test of sphericity was significant (*p* < 0.05), confirming the adequacy of factor structure for the descriptive purpose of this study.

### 2.3. Anthropometric Measurements and Laboratory Testing

Standardized physical examinations were performed on all consenting students. Anthropometric measurements included height and weight. Screening for malnutrition and for overweight/obesity was performed according to the national health industry standards WS/T 456-2014 [19] and WS/T 586-2018 [20], respectively. Body mass index (BMI) was calculated using the formula: BMI (kg/m^2^) = weight (kg)/height^2^ (m^2^). Growth retardation was defined using agespecific height thresholds, whereas mild and moderate-to-severe wasting were defined using age-specific BMI thresholds. Overweight and obesity were defined using sex-and age-specific BMI thresholds. Capillary blood samples were analyzed for hemoglobin to evaluate iron status. We used the HemoCue hemoglobin detection method to measure the Hb in whole blood. For hemoglobin quality control, each site used three control samples (one blind). Pre-analysis quality control required dedicated slides for each device (five consecutive measurements for approval) and 20 blind measurements per device before field work. The diagnostic criteria for anemia established by the WHO were used as the reference and correct the altitudes for different counties. Anemia was defined based on hemoglobin (Hb) thresholds specific to age and sex: <115 g/L for children aged 5–11 years; <120 g/L for children aged 12–14 years and for girls aged 15 years; and <130 g/L for boys aged 15 years.

### 2.4. Statistical Analysis

Categorical variables were summarized as counts (*n*) and percentages (%), while continuous variables were expressed as mean ± standard deviation (SD). All analyses were conducted using R version 4.4.1. Given the large sample size (n > 4000), parametric methods (independent-samples *t*-test or one-way ANOVA) were used for continuous outcomes without prior normality testing, as the central limit theorem ensures robustness. Chi-square tests were used for categorical outcomes. Bonferroni was used for correcting multiple comparisons. A two-tailed *p*-value < 0.05 was considered statistically significant. In our dataset, only one participant had missing data for height or weight and was therefore excluded from the analyses. All other participants had complete data for the key variables.

## 3. Results

### 3.1. Characteristics of the Study Participants

The demographic composition of the study population remained stable between 2014 and 2023, while the distribution of nutritional status shifted markedly. The baseline characteristics of participants aged 6–15 years in 2014 (n = 4388) and 2023 (n = 4231) were presented in Table 1. Age distribution was comparable between the two survey years No significant sex differences were observed overall. While sex ratios were generally consistent across counties, Yunyang County showed a modest increase in the proportion of boys in 2023. Geographic representation remained stable, with each of the six county-level cities accounting for 15% to 18% of the total sample and showing no significant annual variation. Approximately two-thirds of the children were primary school students (69.2% in 2014 vs. 66.9% in 2023). Compared with 2014, the rates of undernutrition and anemia decreased in 2023, while the rate of overweight and obesity increased.

### 3.2. Satisfaction and Nutritional Knowledge Among Students and Parents Under the NIP

Student and parent satisfaction with school meals improved substantially over the nine-year period, accompanied by significant gains in nutritional knowledge (Table 2). The proportion of students reporting that “Very satisfied” rose, while the proportion of “Dissatisfied” ratings declined among both students and parents. Perceived deficiencies in school meals shifted from limited variety and poor taste to predominantly insufficient dish diversity. Compared with 2014, self-reported food waste decreased in 2023, with a higher proportion of students “Always can finish” their meals. The belief that school food was more nutritious than home meals nearly doubled, and the number of parents holding this belief also increased slightly. Meanwhile, both students and parents became more confident in the hygiene standards of school food.

Across all grades and regions, nutritional knowledge scores for both students and parents increased significantly between 2014 and 2023 (Table S1). Average student scores rose from a range of 3.2–4.3 to 5.4–6.3, and parental scores increased from 4.0–4.2 to 5.6–6.1. The largest improvements were observed in Macheng City (students: +4.3) and Yunxi County (parents: +2.2), indicating consistent program-wide knowledge enhancement.

Furthermore, awareness of ten nutrition-related items improved markedly among both students and parents between 2014 and 2023 (Table S2). The largest gains among students were recorded for “Food sources of vitamins” (+34.6–43.0%), “Sunlight exposure and health” (+20.5–41.4%), and “Calcium-rich foods” (+17.7–43.8%), with similar improvements observed in parents. Recognition of the link between unhealthy eating habits and cancer increased across all grade levels, and knowledge about anemia prevention more than doubled by 2023. Overall, compared with 2014, awareness of unhealthy eating habits had increased in 2023, indicating substantial and consistent nutrition knowledge improvement across grades and respondent groups.

### 3.3. Changes in Height and Weight of Students Under the NIP

Mean height and weight increased at every age for both sexes between 2014 and 2023, indicating sustained improvements in linear and ponderal growth (Tables S3 and S4). Compared with 2014, the average height across all age groups was higher in 2023, consistent with national survey data on Chinese children and adolescents. The increase peaked at 7.2 cm for boys and 6.7 cm for girls around the pubertal growth spurt in 2023, with smoothed values generally above 3% before age 12 and tapering to 1% by age 15, reflecting a sustained upward shift in linear growth.

**Figure 1:**
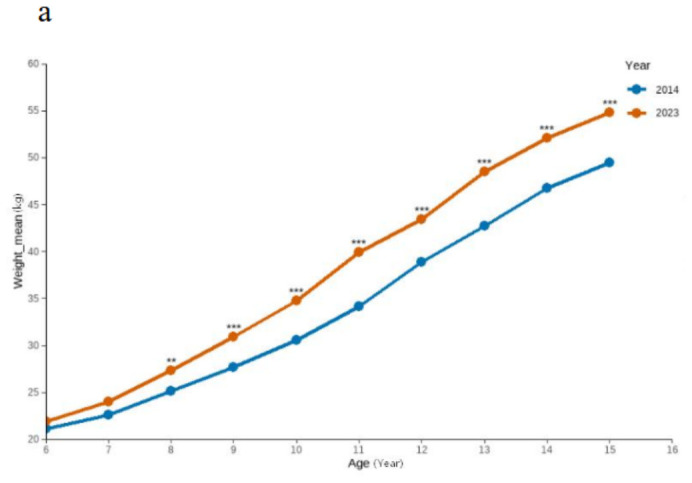

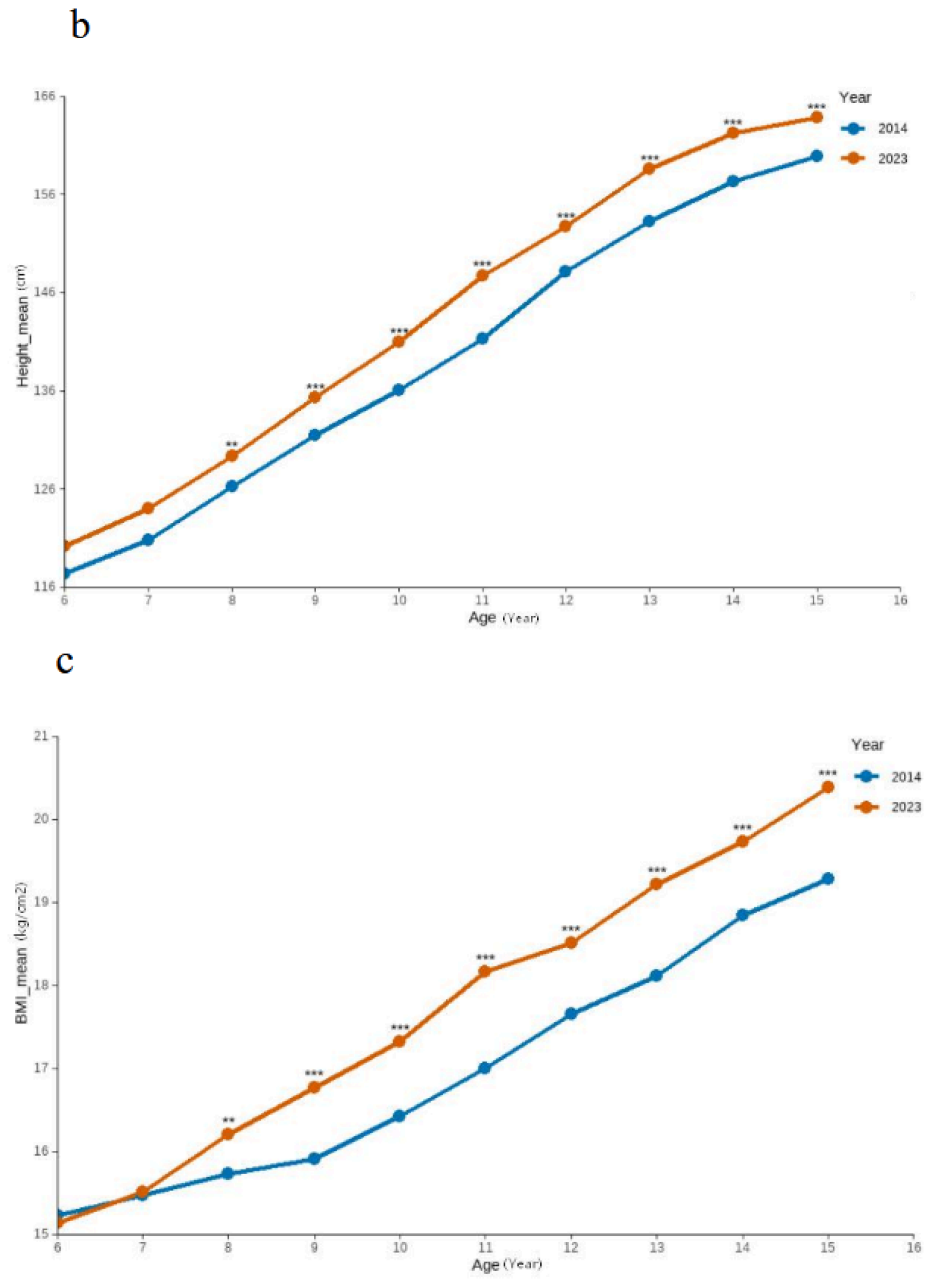
Trends in anthropometric indicators among students under the Nutrition Improvement Program (NIP) in 2014 and 2023. (**a**) Weight; (**b**) height; (**c**) body mass index (BMI). Data are presented as mean values for each age group. *p* values indicate comparisons between 2014 and 2023: ** *p* < 0.01, *** *p* < 0.001.

Furthermore, under the NIP, the rate of weight gain increased in 2023 than in 2014. Mean body weight increased at every age for both sexes, with the largest annual increments occurring at age 15 years in boys and at ages 13 years in girls (Table S5), indicating consistent improvements in ponderal growth. The trends of changes in height, weight, BMI and age were shown in Figure 1.

### 3.4. Changes in the Prevalence of Undernutrition Under the NIP

The prevalence of all indicators of undernutrition declined significantly across all age and sex subgroups from 2014 to 2023 (Table 3). Overall, moderate-to-severe growth retardation fell from 4.1% to 0.6%, mild emaciation from 7.1% to 5.4%, moderate-to-severe emaciation from 5.7% to 3.5%, and total from 16.9% to 9.5%. The results were similar across different age groups. Moreover comparable reductions were observed in both boys and girls, indicating a sustained improvement in nutritional status.

### 3.5. Changes in the Prevalence of Overweight and Obesity Under the NIP

Overweight and obesity increased markedly during the study period, with the largest gains observed among boys and in certain counties. We further quantified the annual percentage-point change in the prevalence of overweight and obesity among boys and girls aged 6–15 years from 2014 to 2023 (Table 4). Overweight prevalence increased from 6.0% to 8.6%, while obesity rose from 3.0% to 7.3%. For boys, overweight prevalence increased by 2.3 percentage points (from 6.6% to 8.9%) and obesity by 5.9 percentage points (from 3.3% to 9.2%). For girls, the corresponding increases were 2.9 percentage points for overweight (from 5.5% to 8.4%) and 2.7 percentage points for obesity (from 2.6% to 5.3%). These trends were consistent with national obesity prevalence data for Chinese children and adolescents.

Table S6 showed that, between 2014 and 2023, the prevalence of undernutrition among students in the NIP fell by 7.4 percentage points, while combined overweight/obesity rose by 6.9 percentage points, resulting in a shift from undernutrition to overnutrition. Normal weight remained stable at 74.1% in 2014 and 74.6% in 2023. The decline in undernutrition was significant in both sexes, all age groups, and most regions (*p* < 0.05), whereas the increase in overweight/obesity was most pronounced among boys (+8.2%), 9–11 years old (+8.8%), and in Yunyang County (+9.1%).

### 3.6. Trends in Anemia Prevalence Among Students Under the NIP

Overall anemia prevalence decreased significantly, but this decline was not uniform across all regions or age groups. Percentiles were calculated to describe the distribution of hemoglobin levels among the study participants (Table S7). Overall, anemia prevalence among students participating in the NIP decreased significantly from 17.1% in 2014 to 12.2% in 2023 (Table 5, *p* < 0.001). The reduction was more pronounced in boys (−5.8%), primary-school pupils (−7.6%), and the 6–8 years old age group (−11.5%). Regionally, Macheng City exhibited the largest decline (−32.0%), whereas Yunyang County showed an unexpected increase (+7.6%). Despite regional inconsistencies, these findings demonstrate an overall improvement in students’ anemia status following the implementation of the NIP.

## 4. Discussion

Over the past few decades, China has experienced rapid economic growth, which has created opportunities to promote human capital development. These opportunities include increased investment in education and training, improved nutrition to enhance health and growth, and the provision of higher-quality medical services to reduce the burden of chronic diseases and the risk of premature death [21]. As the cornerstone of society’s future, children and adolescents have consistently been a core focus of public policy and social research [22,23]. Although the urban–rural gap has shown a gradual narrowing trend in recent years, the issue of uneven resource allocation remains to be addressed.

To improve the nutritional status of rural students and promote educational equity, the Chinese government promulgated and implemented the NIP. Numerous studies have demonstrated that interventions targeting malnutrition in impoverished rural areas—including economic subsidies—benefit the nutritional status, physical fitness, and overall health of children and adolescents [24]. Research has also shown that the NIP positively affects students’ cognitive performance by improving their health conditions, increasing school attendance rates, fostering good study habits, and enhancing educational expectations [25].

In Yunnan Province, the NIP improved growth and development indicators and alleviated vitamin A deficiency among rural students, although no significant changes were observed in persistent anemia or vitamin D levels. Moreover, notable regional differences were reported [9]. During the eight years of NIP implementation in Hainan Province, the growth and development levels of rural students improved significantly. Nevertheless, the growth rate of rural students of the same age still lagged behind the national average. While the incidence of undernutrition decreased, it remained high, and the prevalence of overnutrition increased concurrently [26].

Compared with the national student physical fitness and health survey conducted in 2019 [27,28], height of 2023 Hubei rural students was generally lower. For boys, all age groups except 6 years showed significantly lower height (*p* < 0.05), with Cohen’s d ranging from −0.21 to −0.39. For girls, significant differences were observed at ages 7, 9, 10, 12, and 14 years (*p* < 0.05), with small effect sizes (*d* = −0.21 to −0.24). Overall, children and teenagers in the NIP areas have shown significant improvements in all indicators compared with 2014.

In addition to physical growth, the detection rates of undernutrition and anemia decreased by 7.4 percentage points and 4.9 percentage points, respectively. The anemia rate among girls was higher than that among boys, which was consistent with existing research [29]. This disparity was mainly attributable to physiological characteristics and differential nutritional needs. Childhood anemia remained a widespread public health issue in Asia, seriously affecting children’s growth, cognitive development, and future potential. National data from 2019 showed that the average hemoglobin level of primary and secondary school students in NIP-covered areas was 135.19 g/L, with an anemia rate of 8.7%. The anemia rate was higher among girls (10.0%) than among boys (7.4%), and higher in the western region (9.8%) than in the central region (7.1%) [30]. The relatively higher anemia rate observed in our study may be related to the large number of labor exports from Hubei Province, a high proportion of rural left-behind children, and a relatively large mountainous area.

In addition, the proportion of students satisfied with the nutritious meals increased significantly, and their level of nutritional knowledge also improved, which was largely consistent with existing evidence [6,31]. In parallel with improvements in nutrition knowledge and satisfaction, the prevalence of undernutrition decreased from 16.9% in 2014 to 9.5% in 2023. While this trend was consistent with the program’s objectives, causal attribution to the NIP was not supported by the repeated cross-sectional design. Nevertheless, the rising prevalence of overweight/obesity highlights the need to integrate prevention and early detection mechanisms into future program monitoring.

Nevertheless, the quality of students’ nutritional meals still required improvement. In 2023, in the NIP implementation areas of Hubei Province, 69.0% of primary school students and 48.9% of junior secondary school students still believed that the food provided by the school had “too few varieties.” Moreover, there was a lack of full-time nutrition and health teachers, and students’ nutritional knowledge remained relatively low. Although students’ average nutritional knowledge level improved over the past nine years, awareness of certain nutritional knowledge points—such as “sweet foods and health” and “nutritious beverages”—was still below 50%. Less than 2.0% of students answered all nutrition knowledge questions correctly.

Malnutrition remains common among rural adolescents in China and was associated with perinatal factors, genetics, and economic conditions [32]. Nutritional deficiencies during childhood can have significant and long-lasting negative effects on muscle and bone development, long-term cognitive function, and the risk of chronic diseases [25,33]. Reducing childhood overweight and obesity while promoting linear growth throughout childhood can alleviate the future disease burden on the country [34]. Our findings reveal that compared with the national student physical fitness and health survey (2019), student growth and development in the study population remained relatively suboptimal, with a double burden of malnutrition. The anemia rate among specific subgroups was showing a rapid upward trend. Although direct comparisons are limited by the different survey years, the 2023 Hubei NIP students still fell slightly below the 2019 national rural averages for height and weight. The malnutrition rate among school-aged children in these areas decreased. Over the same period, overweight and obesity prevalence increased. Studies have found that under the NIP, the prevalence of overweight in central and western China rose from 8.0% in 2012 to 10.2% in 2023, while obesity prevalence increased significantly from 3.0% to 8.7% [35]. Our estimates of overweight and obesity were lower, which may be influenced by regional economic development levels and the pace of dietary structure transition. The notable increase in overweight/obesity prevalence observed over the nine-year period reflects both national trends and potential program-specific dynamics. Furthermore, the NIP was associated with greater changes in boys, a finding consistent with previous literature and the Report on the Nutrition and Chronic Disease Status of Chinese Residents (2020) [6].

Several concurrent macro-level factors may have contributed to the observed temporal trends independently of the NIP. Between 2014 and 2023, China experienced rapid economic development, widespread poverty alleviation, improved healthcare accessibility, and a dietary transition toward higher consumption of edible oils, refined grains, and processed foods, alongside reduced physical activity due to sedentary lifestyles. These broader socioeconomic and lifestyle changes likely played a substantial role in the observed decline in undernutrition and the concurrent rise in overweight/obesity. Therefore, the trends reported here should be interpreted as the net result of multiple interacting forces rather than the direct effect of the NIP alone. Future studies collecting individual-level data on household income, dietary intake, and physical activity are needed to statistically disentangle program-specific effects from background secular trends.

Notably, compared with 2014, the anemia rate among students aged 12–15 years in Hubei Province did not decrease significantly in 2023, and in Yunyang County and Enshi City, it increased substantially, by 7.6 and 6.4 percentage points, respectively. Several factors may contribute to these disparities. Firstly, although all counties were classified as poverty-stricken counties, the differences in social and economic conditions among various regions may affect the extent of improvement in the outcomes of nutritional intervention. Yunyang County and Enshi City were located in remote areas, with limited economic development in the past and a scattered distribution of rural populations, which may hinder the fair access to high-quality school meals. Secondly, differences in dietary patterns and food availability across different regions may affect iron intake and its bioavailability, especially the inclusion of animal-based foods in school meals, the use of iron-fortified foods, and the content of iron-absorption inhibitors such as phytates and polyphenols in the meals. Thirdly, regional differences in the implementation of the project, including the nutritional density of meals, staff training, and the intensity of nutrition education, may be the reason why some regions have not fully benefited from the NIP. Fourthly, the increased iron demand during adolescence due to growth spurts and menstrual blood loss (for girls) may not be met by the corresponding increased meal portions. Finally, differences in infection burdens between regions, such as chronic intestinal blood loss caused by hookworm infections, and hemolysis and inflammatory responses caused by infections like malaria, may further exacerbate iron deficiency. Due to the lack of data on dietary intake, project implementation, and infection status in this study, we were unable to directly examine these mechanisms. Future research and project monitoring should include these indicators to guide the adoption of targeted corrective measures in high-risk areas.

The persistent deficiencies in menu quality, food variety, and nutrition education reflect multiple implementation barriers. First, monotonous menus with limited protein sources and fresh vegetables. Second, logistical and infrastructural shortcomings, including inadequate kitchen facilities, untrained catering staff, and weak supervision. Third, nutrition education remains underdeveloped due to the absence of trained personnel and standardized curricula, compounded by low nutrition literacy among students, parents, and school staff. Therefore, supervision efforts should be strengthened to safeguard student nutrition. Schools in NIP implementation areas should be staffed with nutritionists or nutrition advisors to guide the design of balanced menus. Training for cafeteria staff on nutritional health knowledge and related skills should be strengthened, and the use of catering software should be increased. Schools should enhance teachers’ professional knowledge by recruiting qualified personnel or organizing training sessions and should organize various nutrition knowledge education activities for students [17].

Our results may differ from other regions where the NIP had been evaluated. Firstly, Hubei’s central geographic location and mixed rural economy—spanning from relatively affluent plains to impoverished mountainous areas—along with its rice-based dietary pattern (distinct from the wheat-dominated north and seafood-rich coast), variable local implementation of the NIP (e.g., school meal delivery models and infrastructure), and a high proportion of left-behind children dependent on school meals, may collectively modulate the program’s outcomes compared with other regions.

Several limitations of this study should be acknowledged. First, the repeated cross-sectional design, while allowing for the description of temporal trends, does not permit causal inference. Because different students were surveyed in 2014 and 2023, the observed changes cannot be causally attributed to the NIP itself. As discussed above, broader socioeconomic and dietary transitions may have contributed to the observed trends independently of the NIP. Second, we did not collect individual-level socioeconomic data (e.g., household income, parental education, occupation), which were potential unmeasured confounders that could influence child nutritional status. Similarly, data on dietary intake, physical activity, and other lifestyle factors were not available, limiting our ability to explore the underlying mechanisms driving the observed trends. Third, sampling weights were not applied because the sampling fractions were approximately proportional to population sizes across strata. However, the lack of full survey-weighted analysis for the complex sampling design was a limitation, and we acknowledge that the absence of survey-weight adjustment or multilevel modeling may affect variance estimates. Future studies should consider these approaches to account for the nested structure of students within schools and counties. Finally, the study was conducted only in Hubei Province, and its representativeness was therefore limited to this region.

Despite these limitations, the study provided valuable descriptive evidence on long-term nutritional trends among rural schoolchildren in a central Chinese province, offering a basis for future evaluation framework development and targeted policy adjustments. Our findings underscored the critical importance of population stratification in the design and evaluation of large-scale public health and nutrition programs. As emphasized by Chiaramonte [36], stratifying target populations according to demographic, geographic, socioeconomic, and health-related characteristics enables the identification of heterogeneous subgroup needs and facilitates more precise, resource-efficient interventions. Based on the observed trends, we recommended further investment in the NIP, alongside comprehensive descriptive monitoring of implementation outcomes. Such monitoring should identify key implementation challenges and their underlying causes, and pinpoint priority regions and population subgroups that require targeted interventions. Regular surveillance of dietary quality (such as micronutrient deficiencies), physical activity, and obesity-related indicators should be integrated into future evaluations to better address the emerging dual burden of malnutrition.

For boys, who exhibited a higher obesity burden, targeted strategies should emphasize physical activity promotion and healthier food choices both in and out of school. For primary school students, where anemia and undernutrition remain concerns, age-appropriate nutrition education and targeted micronutrient supplementation should be prioritized. For regions with persistent anemia, enhanced investment in diversified meals and iron-rich food supply was warranted, while areas with rapid obesity increases may focus more on energy-balance interventions. NIP could adopt an integrated framework that simultaneously targets all three forms of malnutrition in future. This required strengthening menu diversity to ensure adequate micronutrient intake, maintaining anemia-control efforts, and incorporating obesity prevention measures.

## 5. Conclusions

Between 2014 and 2023, the nutritional health status of students in the surveyed Hubei Province schools improved, while their growth and development still lagged behind to some extent. The primary nutritional concern has now shifted to overnutrition, and the concurrent decline in undernutrition and rise in overweight/obesity highlight an emerging double burden of malnutrition. Given the repeated cross-sectional design, these observed trends cannot be causally attributed to the NIP itself. Nevertheless, the findings suggest that future program efforts should pursue a dual-track strategy: preventing obesity while sustaining anemia and undernutrition reduction, with particular attention to the rapid upward trend in anemia among specific subgroups. Geographically and demographically tailored strategies—accounting for differences in age, sex, and regional economic conditions—are recommended to address the heterogeneous nutritional needs across populations.

## Figures and Tables

**Table 1:** Baseline Characteristics of Participants.

Characteristic	2014				2023							
	Boys (n = 2246)	Girls (n = 2142)	Total (n = 4388)	*P* ^1^	Boys (n = 2148)	Girls (n = 2083)	Total (n = 4231)	*P* ^2^	*P* ^3^	*P* ^4^	*P* ^5^	*P*^5^/Effect Size (95% CI)
age, (years)												
6	85 (3.8)	80 (3.7)	165 (3.8)	0.982	45 (2.1)	54 (2.6)	99 (2.3)	0.287	**0.001**	**0.045**	**<0.001**	−1.4% (−2.1%, −0.7%)
7	231 (10.3)	240 (11.2)	471 (10.8)	0.313	224 (10.4)	237 (11.4)	461 (10.9)	0.327	0.962	0.851	0.835	0.1% (−1.2%, +1.5%)
8	236 (10.5)	245 (11.4)	481 (11.0)	0.312	233 (10.8)	238 (11.4)	471 (11.1)	0.493	0.886	1.000	0.890	0.1% (−1.2%, +1.5%)
9	276 (12.3)	261 (12.2)	537 (12.3)	0.968	241 (11.2)	204 (9.8)	445 (10.5)	0.128	0.275	**0.019**	**0.013**	−1.8% (−3.1%, −0.4%)
10	256 (11.5)	254 (11.9)	510 (11.6)	0.652	248 (11.6)	254 (12.2)	502 (11.9)	0.521	1.000	0.715	0.789	0.3% (−1.1%, +1.6%)
11	271 (12.1)	209 (9.8)	480 (10.9)	**0.015**	252 (11.7)	231 (11.1)	483 (11.4)	0.501	0.619	0.186	0.607	0.5% (−0.9%, +1.8%)
12	214 (9.5)	235 (11.0)	449 (10.2)	0.109	236 (11.0)	225 (10.8)	461 (10.9)	0.839	0.146	0.924	0.326	0.7% (−0.6%, +2.0%)
13	233 (10.4)	234 (10.9)	467 (10.6)	0.571	247 (11.5)	248 (11.9)	495 (11.7)	0.688	0.301	0.302	0.123	1.1% (−0.3%, +2.4%)
14	243 (10.8)	232 (10.6)	475 (10.8)	0.794	255 (11.9)	228 (10.9)	483 (11.4)	0.338	0.356	0.700	0.336	0.6% (−0.7%, +1.9%)
15	201 (8.9)	152 (7.0)	353 (8.0)	**0.032**	167 (7.8)	164 (7.9)	331 (7.9)	0.912	0.238	0.275	0.904	−0.2% (−1.4%, +0.9%)
Education level, n (%)				0.836				0.925	0.123	0.360	**0.012**	
Primary school	1560 (69.5)	1478 (69.0)	3038 (69.2)		1439 (67.0)	1392 (66.8)	2831 (66.9)					Reference
Junior high school	686 (30.5)	664 (31.0)	1350 (30.8)		709 (33.0)	691 (33.2)	1400 (33.1)					2.3% (+0.4%, +4.3%)
Region												
Enshi City	353 (15.7)	343 (16.0)	696 (15.9)	0.666	354 (16.5)	362 (17.4)	716 (16.9)	0.444	0.322	0.133	0.065	1.0% (−0.5%, +2.6%)
Hefeng County	375 (16.7)	398 (18.6)	773 (17.6)	0.108	366 (17.1)	347 (16.6)	713 (16.9)	0.790	1.000	0.125	0.241	−0.7% (−2.4%, +0.8%)
YunYang County	423 (18.8)	347 (16.2)	770 (17.5)	**0.020**	379 (17.6)	312 (15.0)	691 (16.3)	**0.018**	0.266	0.331	0.121	−1.2% (−2.8%, +0.4%)
Yunxi County	349 (15.5)	351 (16.4)	700 (16.0)	0.461	356 (16.6)	354 (17.0)	710 (16.8)	0.724	0.479	0.582	0.336	−0.8% (−0.7%, +2.4%)
Changyang County	372 (16.6)	366 (17.1)	738 (16.8)	0.665	338 (15.7)	348 (16.7)	686 (16.2)	0.399	0.389	0.815	0.417	−0.6% (−2.2%, +1.0%)
Macheng City	374 (16.7)	337 (15.7)	711 (16.2)	0.392	355 (16.5)	360 (17.3)	715 (16.9)	0.520	0.816	0.179	0.434	0.7% (−0.9%, +2.3%)
Weight status, n (%)				**<0.001**				**<0.001**	**<0.001**	**<0.001**	**<0.001**	
Normal	1586 (70.6)	1666 (77.8)	3252 (74.1)		1501 (69.9)	1654 (79.4)	3155 (74.6)					0.5% (−1.3%, 2.3%)
Undernutrition	438 (19.5)	302 (14.1)	740 (16.9)		258 (12.0)	144 (6.9)	402 (9.5)					−7.4% (−8.8%, −6.0%)
Overweight	148 (6.6)	118 (5.5)	266 (6.0)		191 (8.9)	175 (8.4)	366 (8.6)					2.6% (1.5%, 3.7%)
Obesity	74 (3.3)	56 (2.6)	130 (3.0)		198 (9.2)	110 (5.3)	308 (7.3)					4.3% (3.4%, 5.2%)
Anemia condition, n (%)				0.494				**0.005**	**<0.001**	**<0.001**	**<0.001**	
Normal	1875 (83.5)	1761 (82.2)	3636 (82.9)		1918 (89.3)	1796 (86.2)	3714 (87.8)					Reference
Anemia	371 (16.5)	381 (17.8)	752 (17.1)		230 (10.7)	287 (13.8)	517 (12.2)					−4.9% (−6.4%, −3.4%)

*P*^1^ and *P*^2^, *p* value for Boys vs. Girls; *P*^3^, *p* value for Boys (2014) vs. Boys (2023); *P*^4^, *p* value for Girls (2014) vs. Girls (2023); *P*^5^, *p* value for Total (2014) vs. Total (2023). Effect sizes (95% CI) for the total comparison are shown in the rightmost column. We have bolded the items with statistically significant differences.

**Table 2:** Student satisfaction with Nutritious school meals provided under the NIP.

Student Nutritional Meal	Grade 4 of Primary School		Grade 5 of Primary School		Grade 6 of Primary School		Grade 7 of Junior High School		Grade 8 of Junior High School	
	2014	2023	2014	2023	2014	2023	2014	2023	2014	2023
Students										
Degree of satisfaction										
Very dissatisfied	10.8	3.0	9.5	3.0	10.1	3.0	11.0	6.6	10.6	7.8
Dissatisfied	12.9	2.3	13.1	3.5	13.0	2.9	12.7	9.4	14.7	9.2
Neutral	27.2	14.3	29.3	22.2	28.2	18.3	30.1	25.2	36.7	27.3
Satisfied	27.0	21.1	25.3	23.6	26.1	22.4	36.0	32.7	28.6	30.2
Very satisfied	22.2	59.3	22.9	47.7	22.6	53.4	10.2	26.1	9.3	25.6
Disadvantages										
Few varieties	50.2	70.5	54.1	67.6	52.2	69.0	53.2	51.3	43.2	48.9
Not delicious	22.6	11.2	26.7	14.6	24.7	12.9	31.9	27.0	41.5	30.8
High price	8.2	4.4	4.6	0.5	6.4	2.4	4.9	2.2	2.3	1.8
Insufficient quantity	19.0	13.8	14.5	17.4	16.8	15.6	10.0	19.5	13.1	18.4
Remaining situation each time										
Never eats	8.4	0.5	4.8	1.9	6.6	1.2	7.8	2.2	6.2	2.6
Sometimes can finish	38.2	10.3	36.4	15.3	37.3	12.8	36.8	19.1	41.9	19.4
Mostly can finish	28.7	25.3	35.8	33.1	32.2	29.2	40.7	45.2	39.4	42.9
Always can finish	24.7	63.9	22.9	49.8	23.8	56.8	14.7	33.6	12.4	35.1
Compared with food at home										
School food is more nutritious	15.4	28.8	13.3	23.1	14.3	26.0	13.7	11.4	8.9	15.1
Food at home is more nutritious	38.0	12.6	32.4	23.4	35.2	18.0	44.6	27.4	53.3	28.7
About the same	40.3	57.4	46.1	50.5	43.2	53.9	37.8	57.5	32.8	53.6
Not clear	6.3	1.2	8.2	3.0	7.3	2.1	3.9	3.7	5.0	2.7
Cleanliness and hygiene conditions										
Yes	55.7	78.2	48.6	78.7	52.2	78.5	48.5	65.6	42.3	66.8
No	11.0	8.7	9.5	4.9	10.2	6.8	17.2	11.2	15.4	10.5
Not clear	33.3	13.1	41.9	16.4	37.6	14.8	34.4	23.2	42.3	22.7
Parents										
Degree of satisfaction										
Very dissatisfied	7.3	3.9	5.4	2.4	6.3	3.2	6.8	6.8	9.5	8.1
Dissatisfied	9.3	5.9	11.2	3.5	10.2	4.7	12.3	7.2	15.4	9.0
Neutral	41.9	20.0	41.4	21.0	41.6	20.5	45.3	30.3	47.1	29.8
Satisfied	28.4	35.7	30.7	33.0	29.6	34.4	27.0	33.8	21.7	37.2
Very satisfied	13.2	34.5	11.4	40.2	12.3	37.2	8.5	22.0	6.3	15.7
Compared with food at home										
School food is more nutritious	16.3	20.8	12.4	20.5	14.3	20.6	16.2	16.1	12.4	18.2
Food at home is more nutritious	24.0	14.4	23.0	11.2	23.5	12.9	33.8	24.8	38.4	24.4
About the same	50.7	60.1	56.7	63.8	53.7	61.9	44.0	55.1	42.7	53.3
Not clear	9.0	4.6	7.9	4.5	8.5	4.6	6.0	3.9	6.5	4.1
Cleanliness and hygiene conditions										
Yes	50.2	80.0	48.1	80.3	49.1	80.1	54.0	73.6	40.6	71.7
No	9.3	4.2	8.2	6.9	8.7	5.5	10.4	8.7	15.4	8.6
Not clear	40.5	15.9	43.8	12.8	42.2	14.4	35.5	17.6	44.0	19.7

**Table 3:** Change in the prevalence of undernutrition among students under the NIP.

Age, (Years)	2014				2023				Change (Percentage Points)			
	Growth Retardation	Mild Emaciation	Moderate- to-Severe Emaciation	Total	Growth Retardation	Mild Emaciation	Moderate- to-Severe Emaciation	Total	Growth Retardation	Mild Emaciation	Moderate- to-Severe Emaciation	Total
6	3.6	3.6	4.7	12.1	1.0	3.0	5.1	9.1	−2.6	−0.6	0.4	−3.0
7	5.9	6.8	5.3	18.0	1.1	9.1	5.6	15.8	−4.8	2.3	0.3	−2.2
8	6.9	6.0	6.4	19.3	0.8	5.9	5.3	12.0	−6.1	−0.1	−1.1	−7.3
9	5.8	7.1	5.4	18.3	0.4	4.0	2.7	7.1	−5.4	−3.1	−2.7	−11.2
10	6.5	5.9	5.3	17.7	0.6	5.0	5.6	11.2	−5.9	−0.9	0.3	−6.5
11	6.9	8.8	6.5	22.2	0.8	3.1	3.5	7.4	−6.1	−5.7	−3.0	−14.8
12	3.8	8.5	4.7	17.0	0.4	5.4	2.4	8.2	−3.4	−3.1	−2.3	−8.8
13	2.8	7.1	4.9	14.8	1.0	5.7	1.6	8.3	−1.8	−1.4	−3.3	−6.5
14	2.1	7.2	7.2	16.5	0.2	6.4	2.1	8.7	−1.9	−0.8	−5.1	−7.8
15	4.2	7.4	4.8	16.4	0.9	4.8	2.7	8.4	−3.3	−2.6	−2.1	−8.0
Total	4.1	7.1	5.7	16.9	0.6	5.4	3.5	9.5	−3.5	−1.7	−2.2	−7.4
Boys												
6	1.2	3.5	7.1	11.8	2.2	2.2	4.4	8.8	1.0	−1.3	−2.7	−3.0
7	6.5	6.5	6.1	19.1	0.9	8.5	6.3	15.7	−5.6	2.0	0.2	−3.4
8	6.4	4.7	8.1	19.2	0.9	5.2	8.2	14.3	−5.5	0.5	0.1	−4.9
9	7.2	8.0	6.5	21.7	0.0	2.9	4.1	7.0	−7.2	−5.1	−2.4	−14.7
10	8.2	4.7	6.6	19.5	0.8	4.8	7.7	13.3	−7.4	0.1	1.1	−6.2
11	8.9	10.3	7.0	26.2	0.8	3.6	5.2	9.6	−8.1	−6.7	−1.8	−16.6
12	4.2	13.1	7.0	24.3	0.8	8.1	3.4	12.3	−3.4	−5.0	−3.6	−12.0
13	1.3	9.0	6.0	16.3	1.2	9.3	2.4	12.9	−0.1	0.3	−3.6	−3.4
14	2.1	11.9	7.8	21.8	0.4	10.2	3.5	14.1	−1.7	−1.7	−4.3	−7.7
15	7.0	10.0	3.5	20.5	1.2	8.4	3.6	13.2	−5.8	−1.6	0.1	−7.3
Total	5.3	7.9	6.2	19.5	0.8	6.4	4.8	12.0	−4.9	−1.8	−1.7	−8.4
Girls												
6	6.3	3.8	2.5	12.6	0.0	3.7	5.6	9.3	−6.3	−0.1	3.1	−3.3
7	5.4	7.1	4.6	17.1	1.3	9.7	5.1	16.1	−4.1	2.6	0.5	−1.0
8	7.3	7.3	4.9	19.5	0.8	6.7	2.5	10.0	−6.5	−0.6	−2.4	−9.5
9	4.2	6.1	4.2	14.5	1.0	5.4	1.0	7.4	−3.2	−0.7	−3.2	−7.1
10	4.7	7.1	3.9	15.7	0.4	5.1	3.5	9.0	−4.3	−2.0	−0.4	−6.7
11	4.3	6.7	5.7	16.7	0.9	2.6	1.7	5.2	−3.4	−4.1	−4.0	−11.5
12	3.4	4.3	2.6	10.3	0.0	2.7	1.3	4.0	−3.4	−1.6	−1.3	−6.3
13	4.3	5.1	3.8	13.2	0.8	2.0	0.8	3.6	−3.5	−3.1	−3.0	−9.6
14	2.2	2.2	6.5	10.9	0.0	2.2	0.4	2.6	−2.2	0.0	−6.1	−8.3
15	0.7	3.9	6.6	11.2	0.6	1.2	1.8	3.6	−0.1	−2.7	−4.8	−7.6
Total	4.2	5.4	4.5	14.1	0.6	4.2	2.1	6.9	−3.7	−1.3	−2.4	−7.2

**Table 4:** Change in the prevalence of overweight and obesity among students under the NIP.

Age, (Years)	2014			2023			Change (Percentage Points)		
	Overweight	Obesity	Overweight/Obesity	Overweight	Obesity	Overweight/Obesity	Overweight	Obesity	Overweight/Obesity
6	7.3	5.5	12.8	8.1	6.1	14.2	0.8	0.6	1.4
7	6.2	5.3	11.5	7.2	8.0	15.2	1.0	2.7	3.7
8	6.7	4.4	11.1	8.3	9.8	18.1	1.6	5.4	7.0
9	6.5	3.2	9.7	6.5	10.1	16.6	0	6.9	6.9
10	5.9	2.7	8.6	8.6	10.4	19.0	2.7	7.7	10.4
11	6.5	3.8	10.3	11.0	8.3	19.3	4.5	4.5	9.0
12	7.1	1.8	8.9	8.2	5.6	13.8	1.1	3.8	4.9
13	3.0	1.5	4.5	7.9	5.3	13.2	4.9	3.8	8.7
14	5.5	1.5	7.0	9.1	3.7	12.8	3.6	2.2	5.8
15	6.8	0.8	7.6	11.8	3.9	15.7	5.0	3.1	8.1
Total	6.0	3.0	9.0	8.6	7.3	15.9	2.6	4.3	6.9
Boy									
6	8.2	7.1	15.3	11.1	8.9	20.0	2.9	1.8	4.7
7	5.2	6.9	12.1	5.4	12.1	17.5	0.2	5.2	5.4
8	8.5	2.5	11.0	9.0	11.6	20.6	0.5	9.1	9.6
9	6.9	2.5	9.4	7.5	14.9	22.4	0.6	12.4	13.0
10	6.6	3.5	10.1	8.5	12.9	21.4	1.9	9.4	11.3
11	8.1	4.8	12.9	13.1	9.1	22.2	5.0	4.3	9.3
12	8.4	2.8	11.2	10.6	5.1	15.7	2.2	2.3	4.5
13	3.9	1.7	5.6	6.1	6.5	12.6	2.2	4.8	7.0
14	5.8	1.2	7.0	9.4	3.9	13.3	3.6	2.7	6.3
15	5.0	1.5	6.5	9.6	6.6	16.2	4.6	5.1	9.7
Total	6.6	3.3	9.9	8.9	9.2	18.1	2.3	5.9	8.2
Girl									
6	6.3	3.8	10.1	5.6	3.7	9.3	−0.7	−0.1	−0.8
7	7.1	3.8	10.9	8.9	4.2	13.1	1.8	0.4	2.2
8	4.9	6.1	11.0	7.6	8.0	15.6	2.7	1.9	4.6
9	6.1	3.8	9.9	5.4	4.4	9.8	−0.7	0.6	−0.1
10	5.1	2.0	7.1	8.7	7.9	16.6	3.6	5.9	9.5
11	4.3	2.4	6.7	8.7	7.4	16.1	4.4	5.0	9.4
12	6.0	0.9	6.9	5.8	6.2	12.0	−0.2	5.3	5.1
13	2.1	1.3	3.4	9.7	4.0	13.7	7.6	2.7	10.3
14	5.2	1.7	6.9	8.8	3.5	12.3	3.6	1.8	5.4
15	9.2	0.0	9.2	14.0	1.2	15.2	4.8	1.2	6.0
Total	5.5	2.6	8.1	8.4	5.3	13.7	2.9	2.7	5.6

**Table 5:** Changes in the prevalence of anemia among students under the NIP.

Group	2014		2023		Change (Percentage Points)	
	Normal	Anemia	Normal	Anemia	Normal	Anemia
Gender						
Boys ***	83.5	16.5	89.3	10.7	5.8	−5.8
Girls ***	82.2	17.8	86.2	13.8	4.0	−4.0
Education level						
Primary school	83.4	16.6	91.0	9.0	7.6	−7.6
Junior high school	81.6	18.4	81.2	18.8	−0.4	0.4
Age						
6–8 ***	79.4	20.6	90.9	9.1	11.5	−11.5
9–11 ***	86.2	13.8	92.8	7.2	6.6	−6.6
12–15 ***	82.2	17.8	81.9	18.1	−0.3	0.3
Region						
Enshi City	82.7	17.3	76.3	23.7	−6.4	6.4
Hefeng County	86.4	13.6	91.5	8.5	5.1	−5.1
Yunyang County	90.4	9.6	82.8	17.2	−7.6	7.6
Yunxi County	83.4	16.6	92.3	7.7	8.9	−8.9
Changyang County	92.0	8.0	90.8	9.2	−1.2	1.2
Macheng City	61.0	39.0	93.0	7.0	32.0	−32.0
Total ***	82.9	17.1	87.8	12.2	4.9	−4.9

Compared with 2014, *** *p* < 0.001.

## Data Availability

The original contributions presented in this study are included in the article/Supplementary Material. Additional raw data are not publicly available due to privacy or ethical restrictions. Further inquiries can be directed to the corresponding author.

## Supplementary Materials

The following supporting information can be downloaded at: https://www.mdpi.com/article/10.3390/nu18132195/s1, Table S1. Changes in nutritional knowledge scores of students and their parents during the NIP; Table S2. Awareness rates of nutrition knowledge among students and parents under the NIP; Table S3. Height growth and development status of students under the NIP; Table S4. Changes in students’ height (cm) under the NIP; Table S5. Changes in students’ weights (kg) under the NIP; Table S6. Changes in the nutritional status composition of students under the NIP; Table S7. The percentile values of hemoglobin (in g/L) for students aged 6 to 15 under the NIP in 2014 and 2023.

## Abbreviations

BMI	Body Mass Index
CNY	Chinese Yuan
WHO	World Health Organization
NIP	Nutrition Improvement Program
