# Supplementary material for: Assessment of Nutritional Trends and Program Implementation Under the Nutrition Improvement Program
Source: Nutrients. 2026 Jul 6;18(13):2195. doi: 10.3390/nu18132195 (PMC13364250; doi:10.3390/nu18132195)
Supplement: Supplementary file 1 [file nutrients-18-02195-s001.zip › nutrients-4364233-supplementary.pdf]

Table S1. Changes in nutritional knowledge scores of students and their parents during the NIP

Grade	2014		2023		Increasing range	
	Mean ± SD	Median	Mean ± SD	Median	Mean	Median
**Students**						
Grade 1 of primary school	3.2 ± 1.8	3.0	6.0 ± 2.3	6.0	2.8	3.0
Grade 2 of primary school	3.7 ± 1.8	3.0	5.4 ± 2.0	6.0	1.7	3.0
Grade 7 of junior high school	4.2 ± 1.8	4.0	6.2 ± 2.0	6.0	2.0	2.0
Grade 8 of junior high school	4.3 ± 1.8	4.0	6.3 ± 1.9	6.0	2.0	2.0
Region						
Enshi City	3.1 ± 1.5	3.0	4.9 ± 2.0	5.0	1.8	2.0
Hefeng County	3.9 ± 1.9	4.0	4.9 ± 2.0	5.0	1.0	1.0
YunYang County	3.7 ± 1.8	4.0	5.7 ± 1.8	5.0	2.0	1.0
Yunxi County	4.1 ± 1.9	4.0	6.8 ± 1.5	7.0	2.7	3.0
Changyang County	4.7 ± 1.9	4.0	5.6 ± 1.9	6.0	1.9	2.0
Macheng City	3.7 ± 1.8	4.0	8.0 ± 1.2	8.0	4.3	4.0
**Parents**						
Grade 1 of primary school	4.1 ± 1.9	4.0	5.6 ± 2.1	6.0	1.5	2.0
Grade 2 of primary school	4.0 ± 1.9	4.0	5.6 ± 2.0	6.0	1.6	2.0
Grade 7 of junior high school	4.2 ± 1.9	4.0	5.9 ± 1.9	6.0	1.7	2.0
Grade 8 of junior high school	4.1 ± 2.0	4.0	6.1 ± 1.8	6.0	2.0	2.0
Region						
Enshi City	3.5 ± 1.7	3.0	5.0 ± 2.1	5.0	1.5	2.0
Hefeng County	3.5 ± 1.9	3.0	5.6 ± 2.0	6.0	2.1	3.0
Yunyang County	3.8 ± 1.7	4.0	5.5 ± 1.9	6.0	1.7	2.0
Yunxi County	4.4 ± 1.9	4.5	6.6 ± 1.6	7.0	2.2	2.5
Changyang County	4.8 ± 1.8	5.0	6.4 ± 1.8	7.0	1.6	2.0

Macheng City	4.3 ± 2.1	4.0	5.7 ± 2.0	6.0	1.4	2.0

Table S2. Awareness rates of nutrition knowledge among students and parents under the NIP

Nutrition knowledge	Grade 4 of primary school		Grade 5 of primary school		Grade 7 of junior high school		Grade 8 of junior high school	
	2014	2023	2014	2023	2014	2023	2014	2023
**Students**								
Unhealthy eating habits (general)	71.3	91.7	75.5	93.1	86.4	96.2	88.5	97.5
Milk and health	36.4	42.1	38.5	45.2	31.4	64.4	43.7	55.3
Food sources of vitamins	16.9	61.9	13.7	48.3	27.0	60.0	19.9	59.5
Sunlight Exposure and Health Outcomes	25.5	66.9	33.3	53.8	34.5	63.4	35.7	71.8
Calcium-Rich Foods	41.4	85.2	49.3	79.5	71.4	89.1	67.8	84.8
Sweets and health Implications	33.3	41.9	40.3	35.1	33.7	40.0	40.9	53.9
Nutrient-Dense Beverages	19.5	50.6	23.9	40.4	18.9	19.0	22.8	22.5
Unhealthy eating habits (cancer-related)	27.4	57.9	36.0	45.6	31.4	54.6	32.6	49.9
How to prevent iron-deficiency anemia	21.5	44.6	26.8	50.4	31.8	62.1	30.6	65.1
Salt and health	30.5	61.5	31.4	52.7	54.2	69.0	52.2	73.3
**Parents**								
Unhealthy eating habits (general)	25.2	30.9	32.2	45.2	30.8	54.3	34.5	53.9
Milk and health	84.3	89.5	80.8	93.1	78.3	94.1	82.9	95
Food sources of vitamins	25.9	60.0	23.5	60.0	31.2	57.4	21.0	60.5
Sunlight Exposure and Health Outcomes	31.7	61.6	34.3	53.3	29.5	59.1	35.8	68.3
Calcium-Rich Foods	60.1	82	58.6	81.7	65.6	80.5	61.7	81.6

Sweets and health Implications	40.5	45.9	42.4	34.1	42.8	47.2	42	52.2
Nutrient-Dense Beverages	25.6	26.6	22.8	24.2	19.6	22.2	22.5	19.2
Unhealthy eating habits (cancer-related)	34.5	50.7	33.7	46.7	34	48.2	28.1	48.6
How to prevent iron-deficiency anemia	31.7	51.6	29.6	60.7	34.2	62.3	29.8	64.9
Salt and health	46.1	65	38.4	56.3	51.7	66.9	52.7	70.1

Table S3. Height growth and development status of students under the NIP.

Age, (years)	2014					2023				
	**Mean (cm)**	**Standard Deviation (cm)**	**Annualized change (cm)**	**Annual growth rate (%)**	**Smoothing value (%)**	**Mean (cm)**	**Standard Deviation (cm)**	**Annualized change (cm)**	**Annual growth rate (%)**	**Smoothing value (%)**
Boys										
6	118.7	5.7	2.4	2.0	/	121.1*	5.2	3.3	2.7	/
7	121.1	6.4	5.6	4.6	3.7	124.4***	6.1	5.0	4.0	3.9
8	126.7	6.8	4.7	3.7	3.9	129.4***	6.0	6.0	4.6	4.3
9	131.4	6.4	4.5	3.4	3.5	135.4***	6.0	5.2	3.8	4.2
10	135.9	6.9	4.9	3.6	3.9	140.6***	6.7	6.2	4.4	4.2
11	140.8	7.7	6.8	4.8	4.5	146.8***	7.5	6.1	4.2	4.4
12	147.6	7.9	6.7	4.5	4.3	152.9***	8.4	7.5	4.9	4.4
13	154.3	8.0	4.9	3.2	3.2	160.4***	8.4	6.0	3.7	3.3
14	159.2	8.0	3.1	1.9	1.8	166.4***	7.2	1.6	1.0	1.4
15	162.3	7.8	/	/	/	168.0***	6.4	/	/	/
Girls										
6	116.0	6.9	4.4	3.8	/	119.5**	5.5	4.1	3.4	/
7	120.4	6.1	5.3	4.4	4.3	123.6***	6.9	5.8	4.7	4.3
8	125.7	7.2	5.8	4.6	4.3	129.4***	6.2	5.7	4.4	4.5
9	131.5	6.3	4.9	3.7	4.0	135.1***	6.4	6.3	4.7	4.7
10	136.4	7.4	5.5	4.0	4.1	141.4***	7.5	7.2	5.1	4.4
11	141.9	7.9	6.7	4.7	4.0	148.6***	7.0	4.0	2.7	3.3
12	148.6	6.9	3.5	2.4	2.9	152.6***	6.3	4.2	2.8	2.2
13	152.1	6.4	3.1	2.0	1.9	156.8***	6.2	0.7	0.4	1.2
14	155.2	5.6	1.8	1.2	1.1	157.5***	5.5	2.1	1.3	0.8
15	157.0	5.9	/	/	/	159.6***	6.3	/	/	/

Compared with 2014, *$P<0.05$, **$P<0.01$, ***$P<0.001$. The age at the beginning and the end were missing the data point before or after, so they were left blank. “Smoothed values” referred to the smoothed annual growth rate, which was derived from the raw annual increments to provide a more biologically plausible and stable growth trajectory across age groups.

Table S4. Changes in students' height (cm) under the NIP

Age, (years)	Boys				Girls			
	2014	2023	increase amplitude	Annualized change (cm/year)	2014	2023	increase amplitude	Annualized change (cm/year)
6	118.7	121.1*	2.4	0.3	116.0	119.5**	3.5	0.4
7	121.1	124.4***	3.3	0.4	120.4	123.6***	3.2	0.4
8	126.7	129.4***	2.7	0.3	125.7	129.4***	3.7	0.5
9	131.4	135.4***	4.0	0.5	131.5	135.1***	3.6	0.4
10	135.9	140.6***	4.7	0.6	136.4	141.4***	5.0	0.6
11	140.8	146.8***	6.0	0.7	141.9	148.6***	6.7	0.8
12	147.6	152.9***	5.3	0.7	148.6	152.6***	4.0	0.5
13	154.3	160.4***	6.1	0.8	152.1	156.8***	4.7	0.6
14	159.2	166.4***	7.2	0.9	155.2	157.5***	2.3	0.3
15	162.3	168.0***	5.7	0.7	157.0	159.6***	2.6	0.3

Note: Compared with 2014, *$P<0.05$，**$P<0.01$，***$P<0.001$.

Table S5. Changes in students' weights (kg) under the NIP

Age, (years)	Boys				Girls			
	2014	2023	increase amplitude	Annualized change (kg/year)	2014	2023	increase amplitude	Annualized change (kg/year)
6	21.8	23.0	1.2	0.2	20.4	21.0	0.6	0.1
7	23.2	24.8***	1.6	0.2	22.1	23.2**	1.1	0.1
8	25.4	27.7***	2.3	0.3	25.0	27.0***	2.0	0.3
9	27.7	32.0***	4.3	0.5	27.6	29.6***	2.0	0.3
10	31.0	35.1***	4.1	0.5	30.2	34.4***	4.2	0.5
11	34.2	39.8***	5.6	0.6	34.2	39.9***	5.7	0.7
12	38.5	43.2***	4.7	0.6	39.2	43.7***	4.5	0.6
13	43.0	48.9***	5.9	0.7	42.4	48.1***	5.7	0.7
14	47.5	54.0***	6.5	0.8	46.0	49.9***	3.9	0.5
15	50.4	57.2***	6.8	0.8	48.3	52.3***	4.0	0.5

Compared with 2014, *$P<0.05$，**$P<0.01$，***$P<0.001$。

Table S6. Changes in the nutritional status composition of students under the NIP

Group	2014			2023			Change (percentage points)		
	Undernutrition	Normal	Overweight/ Obesity	Undernutrition	Normal	Overweight/ Obesity	Undernutrition	Normal	Overweight /Obesity
Gender									
Boys***	19.5	70.6	9.9	12.0	69.9	18.1	-7.5	-0.7	8.2
Girls***	14.1	77.8	8.1	6.9	79.4	13.7	-7.2	1.6	5.6
Education level, n (%)									
Primary school	18.1	72.0	9.9	10.3	72.6	17.1	-7.8	0.6	7.2
Junior high school	14.0	79.3	6.7	7.7	78.7	13.6	-6.3	-0.6	6.8
Age									
6-8***	17.1	71.5	11.4	13.2	70.4	16.4	-3.9	-1.1	5.0
9-11***	18.2	72.3	9.5	8.5	73.2	18.3	-9.7	0.9	8.8
12-15***	15.5	77.6	6.9	8.2	78.1	13.7	-7.3	0.5	6.8
Region, n (%)									
Enshi City	23.8	69.1	7.1	6.3	78.5	15.2	-17.5	9.4	8.1
Hefeng County	14.4	74.9	10.7	9.5	75.4	15.1	-5.0	0.5	4.4
Yunyang County	15.7	75.3	9.0	8.5	73.4	18.1	-7.2	-1.9	9.1
Yunxi County	18.4	74.9	6.7	13.7	72.2	14.1	-4.7	-2.7	7.4
Changyang County	19.6	67.9	12.5	8.7	73.3	18.0	-10.9	5.4	5.5
Macheng City	9.3	83.4	7.3	10.2	74.7	15.1	0.9	-8.7	7.8
Total***	16.9	74.2	9.0	9.5	74.6	15.9	-7.3	0.4	6.9

Compared with 2014, $*P<0.05$，$**P<0.01$，$***P<0.001$。

Table S7. The percentile values of hemoglobin (in g/L) for students aged 6 to 15 under the NIP in 2014 and 2023

Age	Boys					Girls				
(Year)	P3	P25	P50	P75	P97	P3	P25	P50	P75	P97
2014										
6	97.2	114.2	123.6	131.2	145.3	104.1	114.9	120.4	129.9	144.7
7	101.3	118.5	126.1	132.4	148.0	102.2	116.5	122.7	130.1	144.9
8	100.3	118.9	128.2	138.0	150.9	102.9	118.4	127.5	136.4	150.3
9	98.7	118.9	128.6	136.8	149.4	101.3	120.8	130.3	136.6	152.4
10	101.7	122.1	133.0	140.2	153.5	101.0	123.5	131.3	138.2	154.3
11	101.3	124.5	132.6	140.4	151.8	105.7	124.9	130.9	140.2	149.8
12	107.2	124.6	134.6	140.9	156.9	107.6	123.5	131.7	141.5	155.4
13	114.4	130.0	137.5	144.9	158.3	107.8	123.9	134.4	142.9	156.6
14	112.2	130.9	139.9	150.0	165.6	104.8	124.9	133.8	146.4	161.5
15	110.0	137.0	145.5	153.5	169.1	104.7	124.2	137.1	145.0	160.1
2023										
6	105.0	120.0	128.0	133.0	138.0	109.0	119.0	130.0	135.0	150.0
7	109.0	123.0	129.0	135.5	146.0	109.0	120.0	127.0	133.0	146.0
8	103.0	122.0	130.0	136.0	146.0	110.0	124.0	130.0	135.0	143.0
9	109.0	123.0	129.0	136.0	147.0	110.0	122.0	128.0	134.0	144.0
10	107.0	124.0	131.0	137.0	146.0	114.0	124.0	130.0	137.0	148.0
11	110.0	122.0	130.0	137.0	147.0	110.0	123.0	131.0	138.0	148.0
12	109.0	126.0	134.0	141.0	155.0	104.0	122.0	130.0	137.0	148.0
13	111.0	127.0	137.0	145.0	160.0	106.0	124.0	132.0	139.5	148.0
14	110.0	135.0	145.0	151.0	161.0	102.0	120.0	129.0	139.0	149.0
15	115.0	142.0	148.0	156.0	165.0	107.0	124.0	131.0	139.0	159.0
